# Sevoflurane Alleviates Reperfusion Arrhythmia by Ameliorating TDR and MAPD_90_ in Isolated Rat Hearts after Ischemia-Reperfusion

**DOI:** 10.1155/2019/7910930

**Published:** 2019-09-22

**Authors:** Guilong Wang, Dongjun Dai, Hong Gao, Yanqiu Liu, Zijun Wang, Huayu Li, Xiaokui Fu

**Affiliations:** ^1^School of Anesthesiology, Guizhou Medical University, Guiyang, Guizhou, China; ^2^The Third Affiliated Hospital of Guizhou Medical University, Duyun, Guizhou, China; ^3^Department of Anesthesiology, The Affiliated Hospital of Guizhou Medical University, Guiyang, Guizhou, China

## Abstract

**Objective:**

To investigate the effect of sevoflurane on the monophasic action potentials (MAPs) in isolated rat hearts after ischemia-reperfusion.

**Methods:**

Twenty-four healthy SD male rats, weighing 280–320 g, were randomly divided into three groups after successful preparation of a Langendorff isolated heart perfusion model with a stabilization period perfusion of 15 min with Krebs–Henseleit (K–H) fluid (*n* = 8): the control group (group A, continuously perfused with K–H fluid for 105 min), the ischemia-reperfusion group (group B, continuously perfused with K–H fluid for 15 min, and then exposed to 60 min of global ischemia induced by Thomas solution followed by 30 min of reperfusion), and the sevoflurane group (group C, K–H fluid contained 1.0 MAC sevoflurane, and other procedures were same as in group B). Heart rate (HR) and MAPs including time course (MAPD_50_ or MAPD_90_) of the epicardium (Epi) and endocardium (Endo) were recorded at the time of balance perfusion for 15 min (*T*_0_), continuous perfusion for 15 min (*T*_1_), reperfusion for 15 min/continuous perfusion for 105 min (*T*_2_), and reperfusion for 30 min/continuous perfusion for 120 min (*T*_3_), and the transmural dispersion of repolarization (TDR) was calculated. The incidence of arrhythmia, time for restoration of spontaneous heart beat, and duration of arrhythmia were recorded during the period of reperfusion.

**Results:**

HR in group B and group C was lower at *T*_2_ and *T*_3_ than that in group A, while that in group B was significantly lower than that in group A at *T*_2_ and *T*_3_, and HR in group C was higher than that in group B at *T*_2_ and *T*_3_ (*P* < 0.05). There was no difference of TDR in each group at *T*_0_ and *T*_1_ (*P* > 0.05), while TDR in group B was increased at *T*_2_ and *T*_3_ compared with that in group C and group A (*P* < 0.05). TDR in group C was decreased compared with that in group B at *T*_2_ and *T*_3_ (*P* < 0.05), while there was no such difference between group C and group A (*P* > 0.05). The time for restoration of spontaneous heart beat and duration of arrhythmia in group C were shorter than those in group B (*P* < 0.05), while cardiac arrhythmia scores in group B were higher than those in group C (*P* < 0.05). There was no difference of MAPD_50_ in each group (*P* > 0.05). The MAPD_90_ in group B was much longer than that in other groups at *T*_2_ and *T*_3_ (*P* < 0.05), while there was no such difference between group C and group A (*P* > 0.05). The prolonged MAPD_90_ at *T*_2_ and *T*_3_ in group B strikingly differed from that at *T*_0_ and *T*_1_ (*P* < 0.05). Nevertheless, there was no such difference in other groups at different time points (*P* > 0.05).

**Conclusion:**

Sevoflurane alleviates reperfusion arrhythmia induced by myocardial ischemia-reperfusion through the shortening of MPAD_90_ in isolated rat hearts.

## 1. Introduction

Reperfusion arrhythmias (RA) are the main complications of cardiopulmonary bypass (CPB) in heart surgery. Arrhythmias, such as ventricular tachycardia and ventricular fibrillation, can induce hemodynamic disorders and sudden cardiac death [1, 2]. In the study of arrhythmias, monophasic action potentials (MAPs) is an indicator for changes of cardiac vectors of cardiac arrhythmias, which can describe the repolarization phase of cardiac myocytes intuitively and steadily, and MAPs is an important means to observe the electrical activity of cardiac myocytes [3, 4]. The action potential duration recorded by the single-phase action potential synchronous recording technique is closely related to arrhythmia.

Studies have shown that sevoflurane can reduce the incidence of RA [5–7], but it is not clear whether sevoflurane can affect MAPs in ischemia-reperfusion myocardium. This study is aimed to explore the effects of sevoflurane on the MAPs in an isolated rat heart of ischemia-reperfusion by synchronous recording in the endocardium and epicardium of cardiac myocytes.

## 2. Materials and Methods

### 2.1. Animals

Healthy male SD rats weighing 280 to 320 g were kept in standard transparent polyethylene cages in a light cycle (darkness of 12 h–light of 12 h) in an animal room at 22°C. They had free access to water and food until the time of test. All experiments were approved by the Institutional Animal Care and Use Committee of Guizhou Medical University, and conducted in accordance with the Guide for the Care and Use of Laboratory Animals.

### 2.2. Preparation of Krebs–Henseleit Solution

Krebs–Henseleit (K–H) fluid and Thomas solution was freshly prepared before each test. The components of the solution (mmol/l) included sodium chloride (118.5), sodium bicarbonate (25), potassium chloride (4.8), magnesium sulfate (1.2), D-glucose (12), potassium dihydrogen phosphate (1.2), and calcium chloride (1.7). Thomas solution (g/l) contained sodium chloride (6.44), potassium chloride (1.2), magnesium chloride (1.52), sodium bicarbonate (0.83), and calcium chloride (0.14). All of these components were dissolved in distilled water, but calcium chloride was added after all the ingredients to prevent precipitation.

### 2.3. Surgical Procedure for Isolated Heart Preparation

The animals were pretreated with intraperitoneal injection of 300 IU heparin and then anaesthetized by sodium pentobarbital (50–60 mg/kg). After sacrifice of the animals, their hearts were excised rapidly and mounted on a nonrecirculating Langendorff apparatus under a constant pressure of 80 mmHg at 37°C and perfused with K–H solution that previously equilibrated with 95% O_2_ and 5% CO_2_ (pH = 7.4).

### 2.4. Study Protocols

In preparation of the hearts as well as the Langendorff setup, we followed the methods of Haghi et al. [8]. The rat hearts were randomly divided into three groups after successful preparation of the Langendorff isolated heart perfusion model and 15 min balanced perfusion of K–H fluid. In the control group (group A), K–H fluid was under continuous perfusion for 105 min. In the ischemia-reperfusion group (group B), K–H fluid was stopped after continuous balanced perfusion for 15 min and cardiac arrest of global ischemia was induced for 60 min with the perfusion of Thomas solution (4°C, 20 ml/kg), while the heart was protected by the low temperature Thomas solution (4°C) around it. Reperfusion of Thomas solution (4°C, 10 ml/kg) was performed for 30 min, and the heart was resuscitated by the perfusion of K–H fluid for 30 min. In the sevoflurane group (group C), K–H fluid contained 1.0 MAC sevoflurane, and other procedures were same as in group IR. The K–H fluid was equilibrated with sevoflurane using Vapor2000 with an air bubbler. The concentration of sevoflurane was also measured in the buffer before entering the aortic cannula using a volatile anesthetic gas monitor.

### 2.5. MAPs Recording

MAPs was recorded by a custom-made electrode, which could record the MAPs of the epicardium (Epi) and endocardium (Endo) simultaneously. The MAPs signals were amplified and recorded with a biological signal collecting and processing system. HR and MAPs including time course (MAPD_50_ or MAPD_90_) of endocardium and epicardium were recorded at the time of balance perfusion for 15 min (*T*_0_), continuous perfusion for 15 min (*T*_1_), reperfusion for 15 min/continuous perfusion for 105 min (*T*_2_), and reperfusion for 30 min/continuous perfusion for 120 min (*T*_3_), and transmural dispersion of repolarization (TDR) was calculated. The incidence of arrhythmia, time for restoration of spontaneous heart beat, and duration of arrhythmia were recorded during the period of reperfusion.

### 2.6. Arrhythmias Analysis

Based on the Lambeth conventions [9], the MAPs were analyzed to determine the number of single ectopic beats, salvos (couplets and triplets), ventricular tachycardia (VT), the total number of ventricular ectopic beats (VEBs), incidence, and duration of VT during the 30 min of reperfusion time.

### 2.7. Statistical Analysis

Statistical analyses were performed using the SPSS18.0 software. Except for the arrhythmia scores, the other data are expressed as mean ± SD after test for normality. The comparison between groups was carried out by using one-way analysis of variance. Pairwise comparison was carried out using the LSD method. The *X*^2^-test was used for enumeration data, and values of *P* < 0.05 were considered statistically significant.

## 3. Results

### 3.1. Effects of Sevoflurane on Heart Rate (HR) and Transmural Dispersion of Repolarization (TDR) in I/R Rats

HR in group B and group C was lower at *T*_2_ and *T*_3_ than that in group A, while it in group B was significantly lower than that in group A at *T*_2_ and *T*_3_, and HR in group C was higher than that in group B at *T*_2_ and *T*_3_ (*P* < 0.05, Table 1). There was no difference of TDR in each group at *T*_0_ and *T*_1_ (*P* > 0.05), while TDR in group B was increased at *T*_2_ and *T*_3_ compared with that in group C and group A (*P* < 0.05). TDR in group C was decreased compared with that in group B at *T*_2_ and *T*_3_ (*P* < 0.05), while there was no such difference between group C and group A (*P* > 0.05, Table 1).

### 3.2. Effects of Sevoflurane on Reperfusion Arrhythmia (RA) in I/R Rats

Arrhythmia scores in group B was higher than that in group C, while the time for restoration of spontaneous heart beat and duration of arrhythmia in group C were shorter than that in group B (*P* < 0.05, Table 2).

### 3.3. Effects of Sevoflurane on MAPs in I/R Rats

There was no difference of MAPD_50_ in each group (*P* > 0.05, Table 3). The MAPD_90_ in group B was much longer than that in other groups at *T*_2_ and *T*_3_ (*P* < 0.05), while there was no such difference between group C and group A (*P* > 0.05). The prolonged MAPD_90_ at *T*_2_ and *T*_3_ in group B strikingly differed from that at *T*_0_ and *T*_1_ (*P* < 0.05). Nevertheless, there was no such difference in other groups at different time points (*P* > 0.05, Table 3).

## 4. Discussion

Langendorff isolated perfused heart model, which may reveal pathophysiological changes in the heart more accurately compared to tissue and cells and eliminate the negatives of neurohumoral factors and the preload and after load of heart [10–12], was used in this study for a favorable assessment for the MAPs on ischemia-reperfusion isolated heart by sevoflurane.

Our results showed that sevoflurane could shorten MAPD_90_ of ischemia-reperfusion myocardium and decrease TDR and the incidence of reperfusion arrhythmias and the time for restoration of spontaneous heart beat and duration of arrhythmia. Lee showed sevoflurane can prolong the QT interval and QTc interval [13], and Gong found that sevoflurane can lengthen APD of single cardiomyocyte under normal physiological conditions [14]. While our study revealed that MAPD_90_ prolonged after ischemia-reperfusion and sevoflurane could shorten it, these all suggested that sevoflurane directly played a role in ameliorating the myocardial repolarization in isolated rat hearts after ischemia-reperfusion.

In the study of arrhythmia, MAPs, an indicator of vector cardiograph for a population of cells, could record myocardial repolarization intuitively, especially after myocardial depolarization [15, 16]. Therefore, it has been regarded as an effective method for the detection of the electrical activity of cardiomyocytes and pharmacologically electrophysiological exploration [17, 18]. Monophasic action potentials could avoid the heterogeneity from different origins. We ensured that MAPs were derived from the same transmural origin. In this way, the sevoflurane effects on each myocardium layer were investigated apart from the evaluation of myocardial repolarization.

Various ion channel currents are involved in cardiac action potential, but the APD is determined by outward and inward currents [19, 20]. MAPD_50,_ where ICa-L, Ikr, and Iks are involved, largely reflects plateau of action potential [21]. APD could be prolonged when the Ca^2+^ inward current increases, which is liable to cause arrhythmia and early after depolarization [22]. The action potential repolarization could be prolonged when Ikr and Iks are inhibited [23]. Iks, a ventricular myocardium repolarization reserve and a remarkable prolonged APD, will present in appearance of suppressed Iks [24]. MAPD_90_ is a representative of phase 3 where mainly mediated by Ikr and Ik1 [25, 26]. These two are the currents which are responsible for the early stage and late stage of phase 3, respectively [27, 28]. The prolonged MAPD_90_ shows that when inhibited, Ikr and Ik1 slow down K^+^ current rate. TDR reflects the nonuniformity of ventricular myocyte repolarization of myocardial layers. The increased TDR is the basic factor of a variety of malignant arrhythmia, especially polymorphic ventricular tachycardia and torsades de pointes ventricular tachycardia. Therefore, sevoflurane may alter MAPs through influencing icon channels.

## 5. Conclusion

In summary, we found sevoflurane could alleviate reperfusion arrhythmia by ameliorating TDR and MAPD_90_ in isolated rat hearts after ischemia-reperfusion.

## Figures and Tables

**Table 1:** Effects of sevoflurane on heart rate (HR) and transmural dispersion of repolarization (TDR) in I/R rats (*n* = 8, $\bar{x}\pm s$).

Parameters	Group	*T*_0_	*T*_1_	*T*_2_	*T*_3_
HR (beats/min)	A	241.00 ± 4.78	240.00 ± 3.51	240.63 ± 4.84	243.57 ± 5.10
	B	243.12 ± 3.09	241.12 ± 3.09	172.63 ± 5.58^ab^	181.75 ± 7.67^ab^
	C	242.55 ± 2.82	244.25 ± 2.12	231.62 ± 2.92^bc^	230.13 ± 2.23^bc^

TDR (ms)	A	3.81 ± 1.25	3.64 ± 1.88	3.86 ± 0.96	3.47 ± 1.38
	B	3.90 ± 1.16	3.82 ± 1.69	5.91 ± 1.06	6.10 ± 1.53
	C	3.32 ± 1.18	3.58 ± 1.27	3.58 ± 1.31^a^	3.76 ± 1.56^a^

Note: compared with the *T*_0_, ^a^*P* < 0.05; compared with the *T*_1_, ^b^*P* < 0.05; compared with group B, ^c^*P* < 0.05.

**Table 2:** Comparison of time for restoration of spontaneous heart beat, arrhythmia duration, and arrhythmia scores in two groups.

Group	*N*	Time for restoration of heart beat (*s*, $\bar{x}\pm s$)	Arrhythmia duration (min, $\bar{x}\pm s$)	Arrhythmia scores (score, *M* (*Q*))
B	8	23.60 ± 5.16	6.82 ± 1.69	0.50 (2.00)
C	8	12.42 ± 4.78^a^	3.28 ± 1.71^a^	0.00 (1.50)^a^

Note: compared with group B, ^a^*P* < 0.05.

**Table 3:** Comparison of MAPs among three groups at different time points (ms, $\bar{x}\pm s$, *n* = 8).

Parameters	Sites	Group	*T*_0_	*T*_1_	*T*_2_	*T*_3_
MAPD_50_	Epi	A	17.71 ± 1.95	16.64 ± 2.88	16.06 ± 3.76	17.87 ± 2.38
		B	17.50 ± 3.16	16.22 ± 1.68	16.11 ± 2.56	17.10 ± 2.53
		C	17.32 ± 3.16	15.08 ± 2.28	17.08 ± 4.24	17.76 ± 2.56
	Endo	A	18.80 ± 2.19	16.03 ± 0.73	15.34 ± 3.67	16.00 ± 3.58
		B	18.94 ± 0.96	16.43 ± 4.88	15.33 ± 3.97	17.50 ± 3.94
		C	18.17 ± 2.86	15.85 ± 3.05	18.90 ± 3.64	17.89 ± 3.82

MAPD_90_	Epi	A	34.71 ± 3.95	33.64 ± 2.88	33.86 ± 3.76	32.87 ± 2.38
		B	33.50 ± 3.16	34.22 ± 1.68	47.11 ± 2.56	45.10 ± 2.53
		C	34.32 ± 3.16	33.08 ± 2.28	39.08 ± 4.24^a^	37.76 ± 2.56^a^
	Endo	A	39.80 ± 2.19	36.03 ± 0.73	35.34 ± 3.67	36.62 ± 3.58
		B	39.94 ± 2.96	35.43 ± 4.88	47.33 ± 3.97	47.50 ± 3.94
		C	39.17 ± 2.86	35.85 ± 3.05	39.90 ± 3.64^a^	37.89 ± 3.82^a^

Note: compared with group B, ^a^*P* < 0.05.

## Data Availability

The data used to support the findings of this study have been deposited at http://www.anesthscape.com/Home/Content?Id=1728.

## References

[1] Chin K. Y., Qin C., May L., Ritchie R. H., Woodman O. L. (2017). New pharmacological approaches to the prevention of myocardial ischemia- reperfusion injury. *Current Drug Targets*.

[2] Wu M.-Y., Yiang G.-T., Liao W.-T. (2018). Current mechanistic concepts in ischemia and reperfusion injury. *Cellular Physiology and Biochemistry*.

[3] Tse G., Wong S. T., Tse V., Yeo J. M. (2016). Monophasic action potential recordings: which is the recording electrode?. *Journal of Basic and Clinical Physiology and Pharmacology*.

[4] Jeong D. U., Lim K. M. (2018). The effect of myocardial action potential duration on cardiac pumping efficacy: a computational study. *BioMedical Engineering OnLine*.

[5] Gong J.-S., Yao Y.-T., Fang N.-X., Li L.-H. (2012). Sevoflurane postconditioning attenuates reperfusion-induced ventricular arrhythmias in isolated rat hearts exposed to ischemia/reperfusion injury. *Molecular Biology Reports*.

[6] Cao J., Xie H., Sun Y. (2015). Sevoflurane post-conditioning reduces rat myocardial ischemia reperfusion injury through an increase in NOS and a decrease in phopshorylated NHE1 levels. *International Journal of Molecular Medicine*.

[7] Zhang F., Chen G., Chen C., Yan M. (2009). Sevoflurane postconditioning converts persistent ventricular fibrillation into regular rhythm. *European Journal of Anaesthesiology*.

[8] Haghi J., Eteraf-Oskouei T., Najafi M. (2018). Effects of postconditioning with fructose on arrhythmias and the size of infarct caused by global ischemia and reperfusion in isolated rat heart. *Advanced Pharmaceutical Bulletin*.

[9] Li D., Lu N., Han J. (2018). Eriodictyol attenuates myocardial ischemia-reperfusion injury through the activation of JAK2. *Frontiers in Pharmacology*.

[10] Salameh A., Keller M., Dähnert I., Dhein S. (2017). Olesoxime inhibits cardioplegia-induced ischemia/reperfusion injury. A study in langendorff-perfused rabbit hearts. *Frontiers in Physiology*.

[11] Watanabe M., Okada T. (2018). Langendorff perfusion method as an ex vivo model to evaluate heart function in rats. *Methods in Molecular Biology*.

[12] Bell R. M., Mocanu M. M., Yellon D. M. (2011). Retrograde heart perfusion: the langendorff technique of isolated heart perfusion. *Journal of Molecular and Cellular Cardiology*.

[13] Lee J.-H., Park Y.-H., Kim J.-T., Kim C.-S., Kim H.-S. (2014). The effect of sevoflurane and ondansetron on QT interval and transmural dispersion of repolarization in children. *Pediatric Anesthesia*.

[14] Gong J. S., Yao Y. T., Fang N. X., Huang J., Li L. (2012). Sevoflurane postconditioning alleviates action potential duration shortening and L-type calcium current suppression induced by ischemia/reperfusion injury in rat epicardial myocytes. *Chinese Medical Journal*.

[15] de Diego C., Pai R. K., Chen F. (2008). Electrophysiological consequences of acute regional ischemia/reperfusion in neonatal rat ventricular myocyte monolayers. *Circulation*.

[16] Wang G., Gao H., Wang Z. (2018). Effects of sevoflurane on arrhythmiaand electrophysiology during the global ischemia-reperfusion in isolated rat hearts. *Journal of Clinical Anesthesiology*.

[17] Zhong Y., Cao P., Tong C., Li X. (2012). Effect of ramipril on the electrophysiological characteristics of ventricular myocardium after myocardial infarction in rabbits. *Journal of Cardiovascular Medicine*.

[18] Wang Y., Chen M.-S., Liu H.-C., Xiao J.-H., Wang J.-L. (2014). The relationship between frequency dependence of action potential duration and the expression of TRPC3 in rabbit ventricular myocardium. *Cellular Physiology and Biochemistry*.

[19] Dorian P., Newman D. (2000). Rate dependence of the effect of antiarrhythmic drugs delaying cardiac repolarization: an overview. *Europace*.

[20] Stengl M., Volders P. G. A., Thomsen M. B., Spatjens R. L. H. M. G., Sipido K. R., Vos M. A. (2003). Accumulation of slowly activating delayed rectifier potassium current (IKs) in canine ventricular myocytes. *The Journal of Physiology*.

[21] Williams B. A., Dickenson D. R., Beatch G. N. (1999). Kinetics of rate-dependent shortening of action potential duration in guinea-pig ventricle; effects of IK1 and IKr blockade. *British Journal of Pharmacology*.

[22] Li G. R., Yang B., Feng J., Bosch R. F., Carrier M., Nattel S. (1999). Transmembranel Ca contributes to rate-dependent changes of action potentials in human ventricular myocytes. *American Journal of Physiology-Heart and Circulatory Physiology*.

[23] Sanson C., Schombert B., Filoche-Rommé B., Partiseti M., Bohme G. A. (2019). Electrophysiological and pharmacological characterization of human inwardly rectifying Kir2.1 channels on an automated patch-clamp platform. *ASSAY and Drug Development Technologies*.

[24] Alvarez-Collazo J., Díaz-García C. M., López-Medina A. I., Vassort G., Alvarez J. L. (2012). Zinc modulation of basal and *β*-adrenergically stimulated L-type Ca^2+^ current in rat ventricular cardiomyocytes: consequences in cardiac diseases. *Pflügers Archiv-European Journal of Physiology*.

[25] Geelen P., Drolet B., Lessard E., Gilbert P., O’Hara G. E., Turgeon J. (1999). Concomitant block of the rapid (IKr) and slow (IKs) components of the delayed rectifier potassium current is associated with additional drug effects on lengthening of cardiac repolarization. *Journal of Cardiovascular Pharmacology and Therapeutics*.

[26] Jost N., Virág L., Bitay M. (2005). Restricting excessive cardiac action potential and QT prolongation: a vital role for IKs in human ventricular muscle. *Circulation*.

[27] Anumonwo J. M. B., Lopatin A. N. (2010). Cardiac strong inward rectifier potassium channels. *Journal of Molecular and Cellular Cardiology*.

[28] Muntean D. M., Kiss L., Jost N., Baczko I. (2014). ATP-sensitive potassium channel modulators and cardiac arrhythmias: an update. *Current Pharmaceutical Design*.

